# Cardiac Structure and Cardiorespiratory Fitness in Young Male Japanese Rugby Athletes

**DOI:** 10.3390/jcdd10010012

**Published:** 2023-01-01

**Authors:** Yoshitaka Iso, Hitomi Kitai, Keiko Ichimori, Megumi Kubota, Miki Tsujiuchi, Sakura Nagumo, Tsutomu Toshida, Toru Yonechi, Mio Ebato, Hiroshi Suzuki

**Affiliations:** 1 Division of Cardiology, Department of Internal Medicine, Showa University Fujigaoka Hospital, Yokohama 227-8501, Japan; 2 Division of Cardiology, Showa University Fujigaoka Rehabilitation Hospital, Yokohama 227-8518, Japan; 3 Department of Clinical Pathology and Laboratory, Showa University Fujigaoka Hospital, Yokohama 227-8501, Japan; 4 Department of Physical Therapy, Showa University School of Nursing and Rehabilitation Sciences, Yokohama 226-8555, Japan; 5 Faculty of Sport Science, Nippon Sport Science University, Yokohama 227-0033, Japan

**Keywords:** athlete’s heart, rugby, young athletes, cardiopulmonary exercise testing

## Abstract

Limited data are available on athlete’s heart for rugby athletes. This study aimed to investigate cardiac structure and its relationship with cardiorespiratory fitness in young Japanese rugby athletes. A prospective cross-sectional study using echocardiography and cardiopulmonary exercise testing (CPET) was conducted on 114 male collegiate rugby players. There was a higher prevalence of increased left ventricular (LV), atrial, and aortic dimensions in the young athletes than that in previously published reports, whereas the wall thickness was within the normal range. Anthropometry and CPET analyses indicated that the forwards and backs presented muscular and endurance phenotypes, respectively. Indexed LV and aortic dimensions were significantly larger in the backs than in the forwards, and the dimensions significantly correlated with oxygen uptake measured by CPET. On the four-tiered classification for LV hypertrophy, abnormal LV geometry was found in 16% of the athletes. Notably, the resting systolic blood pressure was significantly higher in athletes with concentric abnormal geometry than in the other geometry groups, regardless of their field positions. Japanese young athletes may exhibit unique phenotypes of cardiac remodeling in association with their fitness characteristics. The four-tiered LV geometry classification potentially offers information regarding the subclinical cardiovascular risks of young athletes.

## 1. Introduction

Long-term exercise training is associated with a spectrum of morphological and functional adaptations of the heart known as athlete’s heart [1]. However, uncertainty remains regarding the most evident expression of athlete’s heart and differential diagnosis including inherited cardiac diseases. Clinicians need to adopt an individualized approach for the interpretation of cardiac evaluation outcomes in athletes because the manifestations of athlete’s heart are influenced by several factors, including the discipline itself.

Rugby comprises mixed activities involving moderate-to-high isometric and isotonic components within the sports discipline [1]. These components vary according to player field position; there are characteristic jobs performed according to position. The main static component activities are performed by the “forward” players (forwards), and the more dynamic components by the “back” players (backs) [2]. Therefore, rugby athletes should provide an ideal model for the assessment of athlete’s heart. In addition, the recent occurrence of high-profile sudden cardiac death events within the sport [3] suggests the need to investigate this population. However, to date, there has been a limited number of studies on athlete’s heart in rugby players, especially young athletes [4,5,6,7]. It is undetermined whether the manifestation of the athlete’s heart structure relates to player field position in young players. In addition, there are no reports about the overall relationship of cardiac structure with cardiorespiratory fitness in rugby athletes.

Cardiovascular evaluation and care of college student-athletes is gaining increasing attention [8]. Therefore, we conducted a prospective cross-sectional study of cardiac evaluation, including echocardiography and cardiopulmonary exercise testing (CPET), in Japanese male athletes on a collegiate rugby team. The aim of this study was to investigate specific cardiac structure according to the player field position and its relationship with cardiorespiratory fitness. We also explored the significance of left ventricular (LV) geometry analysis, if abnormal geometry was identified.

## 2. Materials and Methods

### 2.1. Participants

Cardiac evaluation was performed for all male freshman athletes (*n* = 120; age, mean 18.4 years) on a rugby team at Nippon Sport Science University from 2015 to 2019 at our institution. The team belonged to the Japanese collegiate rugby union group A. Almost all athletes started their rugby career between 10 and 13 years of age and continued training for competitive rugby teams of their schools and/or communities. More than half of the freshman athletes entered the university on a sports scholarship. The athletes participated in structured exercise training with the college team for >10 h/week including both position-specific and common programs.

This was a prospective cross-sectional study. We excluded three athletes who did not undergo echocardiography for various reasons. Three Polynesian athletes were also excluded because of our focus on Japanese athletes and intention to avoid racial confounding. Ultimately, we evaluated the data of 114 Japanese athletes who underwent echocardiography. None of the athletes had mixed ethnic origins.

The athletes were divided into two groups according to their field position: the forwards, including the scrum positions of the prop, hooker, lock, flanker, and number 8 (*n* = 52), and the backs, including the positions of the half, center, and full backs, and wings (*n* = 62). At the time of cardiac screening, bioelectrical impedance analysis was performed using InBody S10 (InBody Japan Inc., Tokyo, Japan) to determine body composition, including body fat mass and appendicular skeletal muscle mass [9]. Body fat mass index and appendicular skeletal muscle mass index were calculated as mass divided by height squared.

### 2.2. Echocardiography

Comprehensive two-dimensional and Doppler echocardiography were performed using digital echocardiography equipment (TUS-A400, Canon Medical Systems, Tochigi, Japan). Standard measurements were performed in accordance with the guidelines of the American Society of Echocardiography and the European Association of Cardiovascular Imaging [10]. Two-dimensional measurements of the left heart included the LV end-diastolic dimension (LVEDD), LV end-systolic dimension (LVESD), thickness of the LV interventricular septum and posterior wall (IVSth and PWth), LV end-diastolic and end-systolic volumes (EDV and ESV), LV mass (LVM), left atrial (LA) anteroposterior diameter (LAD) and volume (LAV), right atrial diameter and area, and aortic root diameter (AOD) at the sinus of Valsalva. All volumes were indexed to body surface area (BSA). LV ejection fraction was calculated using the biplane-modified Simpson’s method. The relative wall thickness (RWT) was defined as the ratio of the sum of the IVSth and PWth at end-diastole to the LVEDD. The z-score of AOD was calculated as the difference between the observed sinus of Valsalva diameter and the value predicted for age, sex, and BSA as previously described [11]. Mild, moderate, and severe aortic dilatation were defined by z-score values of 2.0–3.0, 3.1–4.0, and >4.0, respectively.

We measured LV global longitudinal strain (GLS), as previously described [12]. In brief, endocardial borders were traced on the end-systolic frame using three apical views (4-, 2-, and 3-chamber views). The software tracked speckles along the endocardial border and myocardium throughout the cardiac cycle. The peak GLS of the LV was computed automatically, and regional data and an average value were generated from six segments for each view.

Phenotypic characterization of LV geometry was based on the 2- and 4-tiered classifications (2TC and 4TC) for LV hypertrophy (LVH) [13]. LVH was defined as LVM/BSA >116 g/m^2^ for both 2TC and 4TC systems. For 2TC, the threshold for concentric remodeling/concentric hypertrophy was set at RWT > 0.42. For 4TC, LV geometry was specified by LV concentricity (LVM/LVEDV^2/3^) and indexed LVEDV. Echocardiographic thresholds for increased concentricity were ≥9.1 g/mL^2/3^ and those for increased LVEDV/BSA were ≥76 mL/m^2^. In 4TC, LV geometry was classified according to the thresholds described above: normal geometry; concentric remodeling; concentric LVH, including dilated and non-dilated forms; and eccentric LVH, including dilated and non-dilated forms.

### 2.3. CPET

CPET was performed using cycle or treadmill ergometers in 110 athletes, as previously described [9,14]. The remaining four athletes did not undergo CPET because of sports-related injuries. The expired gas was collected and analyzed continuously using an AE-310S gas analyzer (Minato Co., Osaka, Japan). After checking the data acceptability of the electrocardiogram, blood pressure, and expired gas analysis parameters at rest, the test was started with 4 min of rest, either sitting on the cycle ergometer or standing on the treadmill. A symptom-limited incremental exercise test was performed. The ramp protocol for the cycle ergometer was 30 W/min. The ramp protocol for the treadmill ergometer was designed to obtain linear increases in oxygen uptake (VO_2_) of 5 mL/min through increases in speed and grade. The protocol was designed using the following equation: VO_2_ (mL/min/kg) = 0.15S^2^ + 0.14SG + 0.45S + 0.40G + 4.23, where S is speed (km/h), and G is grade (%) [15]. Peak VO_2_ was defined as the highest VO_2_ value achieved during peak exercise. The oxygen uptake efficiency slope (OUES) was calculated using a logarithmic regression curve between VO_2_ and ventilation during exercise. OUES/kg was able to assess cardiopulmonary functional capacity in children and adolescents with and without congenital heart disease, even at the submaximal level of exercise [16]. The oxygen pulse reflects the amount of oxygen extracted per heartbeat and provides an estimate of the LV stroke volume changes during exercise. The oxygen pulse at peak was calculated as the ratio of VO_2_ to the heart rate at peak exercise time.

### 2.4. Statistical Analysis

Data were analyzed using commercial software (JMP Pro, version 16.0, SAS Institute Inc., Cary, NC, USA). All data are expressed as mean ± standard deviation unless otherwise indicated. Categorical and continuous variables were compared between the groups using the chi-square test and unpaired *t*-test, respectively. Pearson’s simple linear regression analysis was used to determine correlation coefficients between the two parameters. *p*-values of <0.05 were considered statistically significant.

## 3. Results

### 3.1. Anthropometry and Cardiorespiratory Fitness

Anthropometric analysis demonstrated that the forwards had significantly greater height and larger body weight, body mass index, BSA, appendicular skeletal muscle mass, fat mass index, and % fat mass ratio than the backs (Table 1). In the CPET analysis, resting and peak VO_2_ and OUES/kg were significantly higher in the backs than in the forwards (Table 2). The absolute OUES, oxygen pulse at peak, and systolic blood pressures at rest in the forwards were slightly but significantly higher than those in the backs.

### 3.2. Echocardiographic Findings of LV Cavity, Wall, and Function

**Figure 1:**
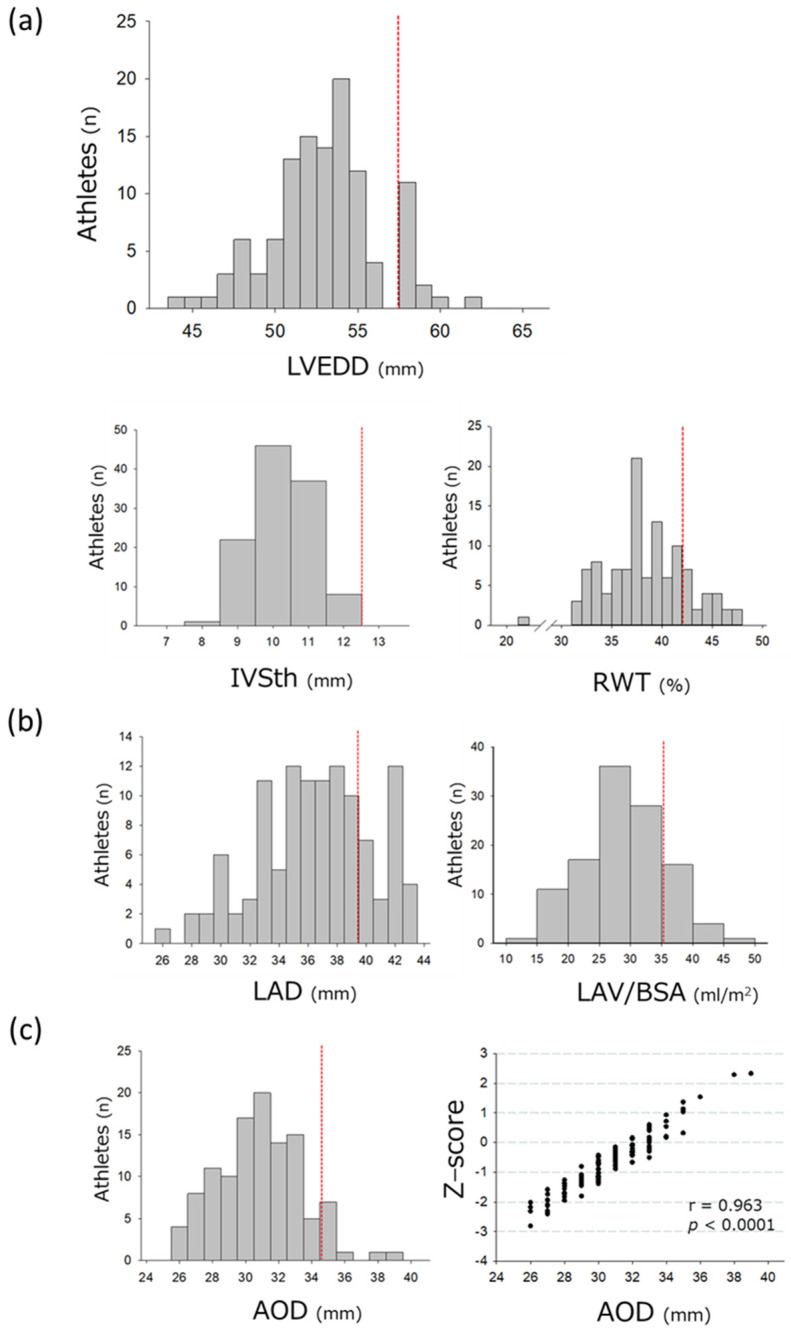
Distributions of dimensions and wall thicknesses of the left heart in young Japanese rugby athletes. Distributions are shown for the (**a**) left ventricle, (**b**) left atria, and (**c**) aorta. Red dotted lines signify the normal upper limit of the general population, according to the guidelines [10]. AOD, aortic root dimension; BSA, body surface area; IVSth, thickness of interventricular septum; LAD, left atrial dimension; LVEDD, left ventricular end−diastolic dimension; RWT, relative wall thickness.

LVEDD, IVSth, and RWT in all athletes were 53.0 ± 3.3 mm, 10.3 ± 0.8 mm, and 0.38 ± 0.04, respectively (Table 3). No athletes were identified with the IVSth or PWth > 12 mm, whereas LVEDD and RWT were abnormally increased in 13.2% of the athletes (LVEDD ≥ 58 mm) and 18.4% of the athletes (RWT ≥ 0.42) according to the reference values of the guideline [10] (Figure 1a).

The echocardiographic parameters were compared between the two player types (Table 3). The absolute values of LVEDD, PWth, LDEDV, LVESV, and LVM were significantly larger in the forwards than in the backs, whereas LVEDD/BSA and LVESD/BSA were significantly larger in the backs than in the forwards. The indexed LVEDV, LVESV, and LVM did not differ between the two groups.

All athletes exhibited normal LV ejection fraction, E/A, and E/e’ (Table 3) without asynergy. The parameters of LV systolic and diastolic function and GLS were not different between the forwards and backs.

### 3.3. Echocardiographic Findings of the Atria and Aortic Root

LAD and LAV/BSA were abnormally increased in 22.8% of the athletes (LAD ≥ 40 mm) and 18.4% of athletes (LAV/BSA ≥ 35), respectively (Figure 1b). The forward athletes exhibited significantly increased LAD compared with the backs (Table 3). The LAV/BSA did not differ between the two groups.

Increased AOD was detected in 8.8% of athletes (AOD ≥ 35 mm) (Figure 1c). There were no athletes with AOD ≥ 40 mm. On the z-score analysis, two athletes showed mild aortic root dilatation. Neither moderate nor severe dilatation was detected in the athletes. AOD was not different between the groups, whereas AOD/BSA was significantly greater in the backs than in the forwards (Table 3). A bicuspid aortic valve without stenosis or regurgitation was found in one athlete with normal AOD and z-scores.

### 3.4. Association of CPET Parameters with Echocardiographic Parameters

We investigated the association between the CPET parameters and indexed values of cardiac structure using Pearson’s linear regression analysis (Table 4). LVEDD/BSA and LVESD/BSA showed weak but significant positive correlations with peak VO_2_ and OUES/kg, respectively. OUES/kg appeared to show a better association than the peak VO_2_. LVEDV/BSA was correlated with OUES/kg and oxygen pulse. The oxygen pulse was significantly correlated with LVM/BSA. The AOD/BSA ratio was significantly associated with peak VO_2_ and OUES/kg. In contrast, LAV/BSA did not exhibit any association with the CPET parameters.

### 3.5. LV Geometry Assessed by the 2TC and 4TC

LV geometry was assessed using the 2TC and 4TC to further elucidate the phenotype of cardiac remodeling in young athletes.

**Figure 2:**
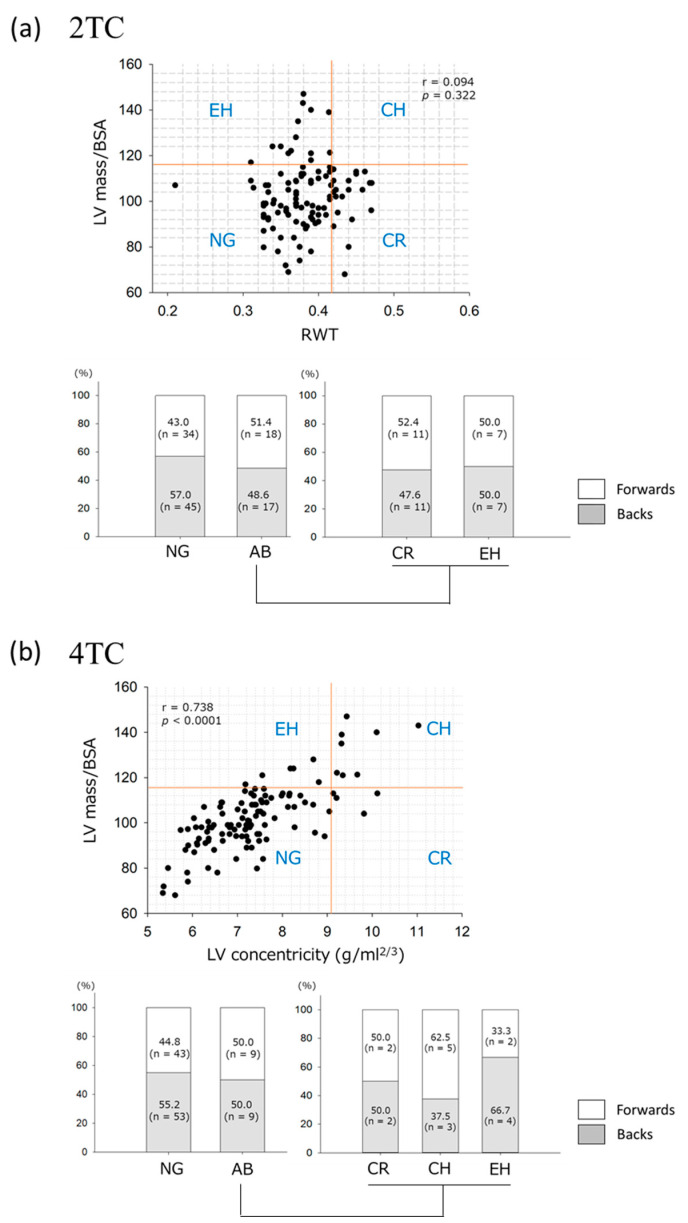
Left ventricular geometry evaluated by (**a**) 2- and (**b**) 4-tiered classifications in young Japanese rugby athletes. The left ventricular geometry is classified according to the thresholds indicated by the red lines. NG, normal geometry; AB, abnormal geometry including concentric remodeling (CR), concentric left ventricular hypertrophy (CH), and eccentric left ventricular hypertrophy (EH); BSA, body surface area.

On the 2TC, the prevalence of each LV geometry category was as follows: normal geometry (*n* = 79) in 69.3% of athletes, concentric remodeling (*n* = 21) in 18.4%, and eccentric LVH (*n* = 14) in 12.3% (Figure 2a). None of the athletes exhibited concentric LVH. In the forwards and backs, the prevalence of normal geometry and abnormal geometry, including concentric remodeling and eccentric LVH, did not differ. The anthropometric and CPET parameters were also comparable regarding the prevalence of the two geometries.

The 4TC was developed to provide a more refined assessment of the geometric patterns of increased LVM. [17] The 4TC in this study demonstrated normal geometry (*n* = 96, 84.2%), concentric remodeling (*n* = 4, 3.5%), concentric LVH (*n* = 8, non-dilated:dilated = 5:3, 7.0%), and eccentric LVH (*n* = 6, non-dilated:dilated = 1:5, 5.3%) (Figure 2b). The prevalence of forwards versus backs did not differ significantly between the normal and abnormal geometry groups or in the results of the 2TC analysis. The anthropometric and CPET parameters, such as peak VO_2_, OUES/kg, and oxygen pulse, were not significantly different among the normal, concentric abnormal (remodeling and LVH, *n* = 12), and eccentric LVH geometry groups, whereas resting systolic blood pressure during CPET was significantly higher in the athletes with concentric abnormal geometry (141.3 ± 17.9 mmHg) than in the athletes with normal (126.7 ± 14.1 mmHg, *p* = 0.004) and eccentric LVH geometry (120.0 ± 6.5 mmHg, *p* = 0.010).

There was a strong relationship between indexed LVM and LV concentricity on the 4TC in these young athletes, whereas no correlation was found between indexed LVM and RWT on the 2TC.

## 4. Discussion

In this study, we investigated specific cardiac structure and cardiorespiratory fitness in young rugby athletes. The data presented here are unique because (i) the variables were derived from two distinct types of athletes, the forwards and backs; (ii) CPET data were involved not only to evaluate cardiorespiratory fitness but also to demonstrate its correlation with cardiac morphology; and (iii) to the best of our knowledge, this is the first study to show LV geometry in young athletes during late adolescence with the use of the 4TC.

***Cardiac structure in the young rugby athletes.*** The present results demonstrated a higher prevalence of increased LV and LA size, and AOD in young rugby athletes compared with the reference ranges of the guidelines [10].

A previous study found that all absolute and indexed structural indices of echocardiography were significantly larger in the professional rugby athletes than in the age-matched sedentary controls [6]. The Japanese Normal Values for Echocardiographic Measurements Project (JAMP) study [18] determined normal values for echocardiographic measurements and the relationships of these parameters with age in a large, healthy Japanese population. Compared with data from the youngest generation (individuals in their 20s) in the JAMP study, our LVEDD and LV wall thickness measurements in late-adolescent athletes in the present study were both larger than in the population (Supplemental Table S1). LVEDV, LVESV, LVM, and LAV were also markedly larger than the variables from the JAMP study, even when divided by BSA.

In a recent study [19] on the Check-up Your Heart Program during the 2015 Gwangju Summer Universiade, LVEDD in Asian athletes (49.7 ± 4.9 mm) was significantly smaller than that in non-Asian athletes (50.5 ± 5.1 mm). The collegiate rugby athletes in our study showed a larger LVEDD (53.0 ± 3.3 mm) than both Asian and non-Asian athletes from a variety of sporting disciplines in the Universiade study. The prevalence of abnormally increased LVEDD in the present study (13.2%) was also somewhat higher than that in the Universiade male athletes (10.4%).

In sports, a mixed discipline is characterized as an activity with alternate phases of intense exercise work and recovery [1]. Typical examples are ball and team activities. In general, athletes belonging to this category exhibit increased LV cavity and modest changes in LV mass and wall thickness [1]. Although rugby belongs to this category, there are also differences in the principal physical requirements of forwards and backs due to their distinct playing roles. Our results demonstrated that the absolute values of LVEDD, LVM, and LAD were significantly larger in the forwards than in the backs. In addition, body size had an important influence on cardiac dimensions. After BSA indexing, LVEDD was significantly larger in the backs than in the forwards, and LVM and LAD did not differ between the two groups. The higher dynamic activity component is performed by the backs. Thus, the athletic structural changes in LV in relation to body size are more likely to occur in the backs than in the forwards. On the other hand, the PWth was significantly larger in the forwards than in the backs. The difference appeared to be attributable to training for the forward job; the predominantly power discipline is characterized by only a mild absolute increase within the upper range of normalcy in LV wall thickness [20]. These findings of collegiate athletes corroborated those of a previous report [4] examining professional rugby players from the French first league. Intriguingly, absolute LVEDD and LVM (59.3 ± 4.7 mm and 224.7 ± 5.4 g, respectively) were larger in the professional athletes than in young Japanese athletes in the present study, whereas the indexed values of LVEDD and LVM (25.9 ± 2.2 mm/m^2^ and 97.1 ± 19.3 g/m^2^, respectively) were smaller.

***Cardiorespiratory fitness and athlete’s heart in rugby athletes.*** Two studies with small numbers of participants were available for evaluating cardiorespiratory fitness using CPET in rugby athletes. Professional rugby players in Italy showed 46.8 ± 4.3 mL/min/kg of a peak VO_2_ (*n* = 15 including both the forwards and bocks) [21]. A study by Scott et al. [22] on professional athletes (*n* = 28) from an English Premiership rugby union club demonstrated a significantly higher peak VO_2_ in backs than in forwards (48.3 ± 2.1 vs. 41.2 ± 2.7 mL/kg/min), similar to our results. The peak VO_2_ difference was likely attributable to the different typical body habitus of forwards and backs as well as position-specific training programs. The forwards are generally taller and heavier players, with greater muscle and fat mass; this could explain their lower peak VO_2_.

However, forwards have shown higher overall exercise intensity than backs during a game [23]. In a study of U.K. rugby players [22], the 3 km run time between the two positions did not significantly differ. Thus, it might be hasty to conclude that the backs are fitter than the forwards. A novel objective indicator of a player’s fitness level assessing the value of position-specific fitness training program is needed. In our study, the absolute OUES was greater in the forwards than in the backs. OUES is an index that integrates the functional capacities of several organ systems, primarily cardiovascular, pulmonary, and musculoskeletal, during exercise [24]. This index could be a candidate to evaluate the fitness of forwards, although further study will be needed to address that.

The endurance capacity of our study population appeared to be relatively lower than that of participants in previous studies [21,22]. We surmise that this occurred because the athletes in our study were in their late adolescence (age 18 years) and still in the physiological growing state.

Although several studies have evaluated the relationship between cardiac structure and cardiorespiratory fitness in athletes [25,26,27,28,29], the results are somewhat controversial. While two studies [25,26] failed to show a correlation between peak VO_2_ and LVEDD or LVM in trained males, other studies [27,28,29] showed good correlations between peak VO_2_ and LVEDD in highly trained male cyclists and accomplished female athletes.

To the best of our knowledge, this is the first study to show the significant correlation in rugby athletes. The present study of young athletes demonstrated that indexed LV dimensions and AOD were significantly larger in the backs than in the forwards, and they were also significantly correlated with the peak VO_2_ and OUES/kg on CPET analysis. Athletes with higher endurance performance likely exhibited not only larger cardiac, but also larger aortic dimensions even after corrections for BSA. Intriguingly, OUES/kg showed a slightly stronger association with the dimensions than peak VO_2_.

***LV geometry evaluated by the 2TC and 4TC in young athletes.*** The 4TC has been developed for a more distinct classification of LV geometry than conventional 2TC, taking three-dimensional information of the LV into account [17]. Compared with the 2TC, the 4TC has demonstrated superior risk stratification for adverse cardiovascular events in the general population [30].

A study [13] by Trachsel et al. demonstrated better discrimination of exercise-induced LVH patterns on the 4TC than on the 2TC in a cohort of middle-aged male Caucasian endurance athletes. This was the only study to evaluate athlete’s heart using the 4TC, although the athletes were at a non-elite level. The present study is the first to assess LV geometry using the 4TC in young competitive athletes in late adolescence. When compared with the previous study, the prevalence of normal geometry on the 4TC was higher in the young athletes (60% vs. 84.2%). Although it is unknown whether this discrepancy is based on age, ethnicity, sport type, or competition level, most young Japanese athletes do not appear to be at risk of adverse cardiovascular events.

Resting systolic blood pressure was significantly higher in the young athletes with concentric abnormal geometry than in those with normal geometry and eccentric LVH. Thus, the 4TC may be better for identifying athletes at risk of hypertension and/or subclinical cardiovascular disorders than the 2TC, although further investigation will be necessary.

***Study limitations.*** There are some limitations to the present study, although it provides detailed information about athlete’s heart in young rugby players. First, the cross-sectional nature of this study limits the understanding of the mechanisms underlying remodeling processes. Second, since our population included a relatively small number of athletes, further studies with larger sample sizes are needed to confirm our results. Third, this study was conducted with a selected cohort of Japanese male athletes. Therefore, our study cannot characterize female athletes. Ethnicity has also emerged as a determinant of athlete’s heart. In a study of rugby athletes, Pacific Islander ethnicity showed greater LV and RV wall thickness and mass than their Caucasian counterparts [5]. Further studies including athletes of a broader race and ethnicity spectrum are necessary for evaluating the impact of East Asian background on athlete’s heart in rugby players. Fourth, we were not able to provide detailed information concerning right ventricular adaptations. Finally, the lack of follow-up did not make it possible to determine the long-term prognosis of abnormal LV geometry. However, there are no reports to date of adverse cardiac events in collegiate life from team doctors and trainers.

## 5. Conclusions

In young rugby athletes, major echocardiographic abnormalities are quite rare, but measurements of LV and LA size and AOD were frequently larger than the guideline reference ranges. Measurements of anthropometry, exercise capacity, and cardiac structure differed between forwards and backs. Changes in left heart dimensions relative to body size occurred more in backs than in forwards. The utility of CPET should also be emphasized. OUES could be useful as a unique parameter to assess fitness and to interpret athlete’s heart in rugby athletes. Finally, compared with the 2TC, the 4TC of LV geometry appeared to contribute to better risk stratification of athletes with LVH.

## Figures and Tables

**Table 1:** Anthropometric data of young Japanese rugby athletes.

	All (*n* = 114)	Forwards (*n* = 52)	Backs (*n* = 62)	*p*-Value
Age (years)	18.4 ± 0.6	18.4 ± 0.5	18.5 ± 0.6	0.266
Ht (cm)	173.3 ± 6.0	175.0 ± 5.7	171.9 ± 6.0	0.005
Wt (kg)	83.1 ± 12.5	92.9 ± 10.5	74.9 ± 7.0	<0.001
BMI (kg/m^2^)	27.6 ± 4.0	30.4 ± 4.0	25.3 ± 2.1	<0.001
BSA (m^2^)	2.0 ± 0.2	2.1 ± 0.1	1.9 ± 0.1	<0.001
Appendicular muscle mass index (kg/m^2^)	9.4 ± 0.8	9.8 ± 0.7	9.1 ± 0.6	<0.001
Fat mass index (kg/m^2^)	5.8 ± 3.0	7.8 ± 3.2	4.1 ± 1.4	<0.001
% Bodyfat (%)	20.0 ± 7.7	24.8 ± 7.9	16.0 ± 4.8	<0.001

BMI, body mass index; BSA, body surface area; Ht, height; Wt, weight.

**Table 2:** Cardiopulmonary exercise testing parameters of young Japanese rugby athletes.

		All (*n* = 110)	Forwards (*n* = 50)	Backs (*n* = 60)	*p*-Value
HR (bpm)	at rest	71.4 ± 8.0	71.6 ± 8.0	71.3 ± 8.0	0.866
	at peak	175.6 ± 15.1	175.6 ± 15.9	175.6 ± 14.6	0.989
SBP (mmHg)	at rest	127.9 ± 15.0	133.5 ± 16.3	123.4 ± 12.1	<0.001
	at peak	194.0 ± 23.4	195.1 ± 25.2	193.0 ± 22.0	0.646
VO_2_ (mL/min/kg)	at rest	4.3 ± 0.6	4.1 ± 0.5	4.5 ± 0.6	0.001
	at peak	42.4 ± 8.0	36.0 ± 7.8	45.2 ± 7.2	<0.001
OUES		3851 ± 603	3978 ± 580	3746 ± 606	0.044
OUES/kg		47.2 ± 8.8	43.5 ± 8.0	50.4 ± 8.2	<0.001
O_2_ pulse at peak (mL/beat)		19.5 ± 2.8	20.2 ± 2.8	19.0 ± 2.7	0.029

HR, heart rate; OUES, oxygen uptake efficiency slope; SBP, systolic blood pressure; VO_2_, oxygen uptake.

**Table 3:** Echocardiographic parameters of young Japanese rugby athletes.

	All (*n* = 114)	Forwards (*n* = 52)	Backs (*n* = 62)	*p*-Value
LVEDD (mm)	53.0 ± 3.3	54.0 ± 3.2	52.1 ± 3.1	0.001
LVEDD/BSA (mm/m^2^)	26.6 ± 2.0	25.6 ± 1.7	27.4 ± 1.9	<0.001
LVESD (mm)	33.2 ± 3.2	33.8 ± 3.1	32.8 ± 3.2	0.098
LVESD/BSA (mm/m^2^)	16.7 ± 1.8	16.0 ± 1.7	17.2 ± 1.7	<0.001
IVSth (mm)	10.3 ± 0.8	10.4 ± 0.9	10.1 ± 0.8	0.068
PWth (mm)	10.1 ± 0.9	10.4 ± 1.0	9.9 ± 0.8	0.002
RWT	0.38 ± 0.04	0.39 ± 0.04	0.38 ± 0.04	0.864
LVEDV (mL)	150.2 ± 26.4	157.6 ± 27.6	143.9 ± 23.8	0.005
LVEDV/BSA (mL/m^2^)	75.0 ± 11.4	74.5 ± 11.9	75.5 ± 11.1	0.633
LVESV (mL)	53.9 ± 10.8	56.6 ± 12.2	51.6 ± 8.9	0.012
LVESV/BSA (mL/m^2^)	27.0 ± 5.0	26.7 ± 5.4	27.1 ± 4.7	0.690
LVM (g)	206.7 ± 34.9	221.1 ± 37.2	194.6 ± 27.8	<0.001
LVM/BSA (g/m^2^)	102.4 ± 14.5	103.4 ± 16.2	101.6 ± 12.9	0.496
LAD (mm)	36.5 ± 3.9	38.1 ± 3.7	35.1 ± 3.5	<0.001
RAD (mm)	41.3 ± 4.9	41.7 ± 4.7	40.9 ± 5.0	0.374
LAV/BSA (mL/m^2^)	28.4 ± 6.4	28.8 ± 7.0	28.0 ± 5.9	0.512
AOD (mm)	30.9 ± 2.6	31.2 ± 2.3	30.6 ± 2.8	0.281
AOD/BSA (mm/m^2^)	15.5 ± 1.6	14.8 ± 1.3	16.1 ± 1.6	<0.001
LVEF (%)	64.4 ± 4.3	64.4 ± 4.6	64.3 ± 4.1	0.940
E/A	2.3 ± 0.5	2.2 ± 0.6	2.3 ± 0.5	0.642
E/e’	6.3 ± 1.3	6.6 ± 1.4	6.1 ± 1.3	0.053
GLS (%)	−17.8 ± 1.4	−17.6 ± 1.3	−17.9 ± 1.4	0.248

AOD, aortic root dimension; BSA, body surface area; GLS, global longitudinal strain; IVSth, thickness of interventricular septum; LAD, left atrial dimension; LAV, left atrial volume; LVEDD, left ventricular (LV) end-diastolic dimension; LVEDV, end-diastolic volume; LVEF, LV ejection fraction; LVESD, LV end-systolic dimension; LVESV, end-systolic volume; LVM, LV mass; PWth, posterior wall thickness; RAD, right atrial dimension; RWT, relative wall thickness.

**Table 4:** Pearson’s correlation analysis between cardiopulmonary exercise testing and echocardiographic parameters.

	Peak VO_2_ (mL/min/kg)		OUES/kg		O_2_ Pulse at Peak (mL/beat)	
	Correlation Coefficient	*p*-Value	Correlation Coefficient	*p*-Value	Correlation Coefficient	*p*-Value
LVEDD/BSA	0.319	<0.001	0.398	<0.001	−0.160	0.096
LVESD/BSA	0.263	0.006	0.353	<0.001	−0.095	0.328
LVEDV/BSA	0.182	0.057	0.198	0.038	0.230	0.016
LVESV/BSA	0.208	0.029	0.141	0.142	0.171	0.076
LVM/BSA	0.144	0.133	0.104	0.281	0.211	0.028
LAV/BSA	−0.091	0.342	−0.051	0.596	0.079	0.413
AOD/BSA	0.263	0.006	0.293	0.002	−0.114	0.238

AOD, aortic root dimension; BSA, body surface area; LAV, left atrial volume; LVEDD, left ventricular (LV) end-diastolic dimension; LVEDV, end-diastolic volume; LVEF, LV ejection fraction; LVESD, LV end-systolic dimension; LVESV, end-systolic volume; LVM, LV mass; OUES, oxygen uptake efficiency slope; O_2_, oxygen; VO_2_, oxygen uptake.

## Data Availability

The data that support the findings of this study are available from the corresponding author upon reasonable request.

## Supplementary Materials

The following supporting information can be downloaded at: https://www.mdpi.com/article/10.3390/jcdd10010012/s1, Table S1: Comparison summary of the present and previous studies regarding the hearts of Japanese athletes.
