# Supplementary material for: Cardiac Structure and Cardiorespiratory Fitness in Young Male Japanese Rugby Athletes
Source: J Cardiovasc Dev Dis. 2023 Jan 1;10(1):12. doi: 10.3390/jcdd10010012 (PMC9861008; doi:10.3390/jcdd10010012)
Supplement: Supplementary file 1 [file jcdd-10-00012-s001.zip › jcdd-2094029-supplementary.pdf]

Table S1: Comparison of echocardiographic parameters between young Japanese rugby athletes and a general Japanese population in their twenties

	Young Japanese rugby athletes (n=114)	Reference data from Japanese participants in their twenties *
LVEDD (mm)	53.0±3.3	49±4
LVEDD/BSA (mm/m^2)	26.6±2.0	27±2
LVESD (mm)	33.2±3.2	31±4
LVESD/BSA (mm/m^2)	16.7±1.8	18±2
IVSth (mm)	10.3±0.8	8±1
PWth (mm)	10.1±0.9	8±1
RWT	0.38±0.04	NA
LVEDV (ml)	150.2±26.4	102±22
LVEDV/BSA (ml/m^2)	75.0±11.4	58±11
LVESV (ml)	53.9±10.8	38±11
LVESV/BSA (ml/m^2)	27.0±5.0	21±6
LVM (g)	206.7±34.9	130±26
LVM/BSA (g/m^2)	102.4±14.5	74±14
LAD (mm)	36.5±3.9	31±4
RAD (mm)	41.3±4.9	37±5
LAV/BSA (ml/m^2)	28.4±6.4	24±8
AOD (mm)	30.9±2.6	29±3
AOD/BSA (mm/m^2)	15.5±1.6	17±2
LVEF (%)	64.4±4.3	NA
E/A	2.3±0.5	2.0±0.5
E/e'	6.3±1.3	4.8±1.5
GLS (%)	-17.8±1.4	NA

AOD, aortic root dimension; BSA, body surface area; GLS, global longitudinal strain; IVSth,

thickness of interventricular septum; LAD, left atrial dimension; LAV, left atrial volume; LVEDD, left ventricular (LV) end-diastolic dimension; LVEDV, end-diastolic volume; LVEF, LV ejection fraction; LVESD, LV end-systolic dimension; LVESV, end-systolic volume; LVM, LV mass; PWth, posterior wall thickness; RAD, right atrial dimension; RWT, relative wall thickness. *, data were derived from the JAMP study (Ref. 18). NA, data was not available.
